# Cell density–dependent nuclear‐cytoplasmic shuttling of SETDB1 integrates with Hippo signaling to regulate YAP1‐mediated transcription

**DOI:** 10.1002/1873-3468.70286

**Published:** 2026-01-19

**Authors:** Jaemin Eom, Jaewoong Jang, Jung Sun Park, Yong‐Kook Kang

**Affiliations:** ^1^ Aging Convergence Research Center (ACRC), Development and Differentiation Research Center Korea Research Institute of Bioscience and Biotechnology (KRIBB) Daejeon South Korea; ^2^ Department of Functional Genomics University of Science and Technology (UST) Daejeon South Korea

**Keywords:** ATF7IP, Hippo pathway, phosphorylation, proteasomal degradation, TEAD

## Abstract

SETDB1, a H3K9 methyltransferase involved in nuclear transcriptional silencing, also localizes to the cytoplasm through unclear mechanisms. Here, we identify cell density as key regulator of SETDB1 subcellular localization and demonstrate its role in modulating the Hippo signaling pathway. Under low‐density culture, SETDB1 distributes between nucleus and cytoplasm, whereas high‐density culture triggers nuclear exclusion and proteasomal degradation. SETDB1 depletion reduces YAP1 phosphorylation and increases nuclear YAP1 accumulation. Transcriptomic analysis of SETDB1 knockout cells revealed upregulation of YAP1–TEAD1 target genes (YTGs). Immunoprecipitation experiments showed that SETDB1 is recruited to YTG promoters via TEAD1 and competes with YAP1 for TEAD1 binding. These findings reveal that SETDB1 regulates Hippo pathway output through YAP1 phosphorylation modulation and competitive transcriptional repression.

## Graphical abstract

At low cell density, SETDB1 and YAP1 accumulate in the nucleus. As cell density increases, the Hippo pathway is gradually activated, and SETDB1 is associated with increased YAP1 phosphorylation. At high cell density, phosphorylated YAP1 is sequestered in the cytoplasm, while SETDB1 becomes polyubiquitinated and degraded by the ubiquitin–proteasome system.

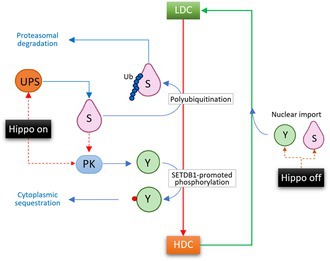

## Abbreviations

**ChIP**, chromatin immunoprecipitation

**DEG**, differentially expressed gene

**HDC**, high‐density culture

**IP**, immunoprecipitation

**LDC**, low‐density culture

**MDC**, medium‐density culture

**SKO**, SETDB1 knockout

**TSS**, transcription start site

**WT**, wild‐type

**YTG**, YAP1‐TEAD target gene

SET domain bifurcated 1 (SETDB1) is a histone methyltransferase that catalyzes H3K9 trimethylation to establish HP1‐dependent heterochromatin and transcriptional repression [1]. SETDB1 regulates its target genes through interactions with transcription factors and corepressors, including KAP1, HDAC1/2, SIN3A/B, and SP3 [2]. Notably, SETDB1 silences endogenous retroviruses (ERVs), which protects genome integrity [3]. It also contributes to small RNA‐guided transcriptional silencing [4]. SETDB1 has additional roles in nuclear architecture, as its depletion disrupts heterochromatin compartmentalization and nuclear integrity [5]. SETDB1 stabilizes PML nuclear bodies [6] and maintains telomeric heterochromatin for the ALT pathway [7], establishing it as a central regulator of genome stability.

Despite its well‐established nuclear role in transcriptional repression, SETDB1 also functions in the cytoplasm. Under certain conditions, particularly overexpression, SETDB1 accumulates in the cytoplasm [8], suggesting an additional regulatory layer for its activity. Cytoplasmic SETDB1 promotes oncogenic signaling through AKT methylation [9], enhances the Warburg effect [10]. It also regulates cytoskeletal organization [11], controls RNA stability [12], and stabilizes the collagen chaperone Serpinh1 [13]. Together, these findings demonstrate cytoplasmic SETDB1 as a multifunctional regulator of non‐nuclear processes.

Emerging evidence indicates that SETDB1 localization is dynamically regulated by signaling pathways (such as Wnt [14] and TGFβ [15]) and mechanical cues. Notably, intracellular mechanical stress from cell geometry and actomyosin contractility via ROCK signaling facilitates nuclear import of SETDB1, while inhibition with Y‐27632 disrupts this process, demonstrating SETDB1 as a mechanosensitive protein [16]. This behavior resembles YAP1/TAZ, key effectors of the Hippo pathway that regulates organ size and cell proliferation by integrating mechanical and biochemical signals such as cell density, actomyosin tension, and matrix stiffness [17]. In the Hippo pathway, MST1/2–LATS1/2 phosphorylate YAP1/TAZ for cytoplasmic retention or degradation, whereas dephosphorylated YAP1/TAZ enter the nucleus to drive growth [17]. These mechanosensitive behaviors of SETDB1 and Hippo components suggest parallel pathways interpreting mechanical cues to influence transcriptional and epigenetic states in tissue homeostasis and cell fate decisions.

SETDB1 subcellular localization is controlled by intrinsic trafficking signals and extrinsic factors. SETDB1 contains nuclear localization signal (NLS) and nuclear export signal (NES) domains, but the dominant NES causes cytoplasmic bias under basal conditions [18, 19]. However, nuclear retention is actively regulated through protein interactions and post‐translational modifications. ATF7IP, a key nuclear adaptor, binds to the SETDB1 NES region to block CRM1‐mediated export and facilitates SETDB1 monoubiquitination at lysine 867, which enhances SETDB1 activity and prevents proteasomal degradation [18, 20]. Together, these findings illustrate a multilayered regulatory system that integrates intrinsic motifs with active stabilization mechanisms.

Building on our previous work on SETDB1 nucleocytoplasmic trafficking and its potential role as a mechanoresponsive regulator intersecting with Hippo signaling, this study investigates the interplay between SETDB1 and the Hippo pathway. Through this investigation, we aim to uncover a novel interface between mechanosensitive signaling and epigenetic control, with broader implications for tissue homeostasis and cancer progression.

## Materials and methods

### Vector construction

Full‐length human YAP1 (NM_001130145.3) and TEAD1 (NM_021961.6) cDNAs were amplified from HEK293T cells by PCR and cloned into pcDNA3.1 expression vectors using restriction enzyme digestion and ligation. The ligation products were transformed into DH5α competent cells, purified using the Plasmid Mini Prep Kit (Biofact, Daejeon, Republic of Korea), and verified by Sanger sequencing. Primer sequences are listed in Table S1.

### Cell culture, transfection, MG132 and leptomycin B treatment

HEK293T (RRID: CVCL_0063) cells were obtained from the Korean Cell Line Bank (KCLB; Seoul, Republic of Korea) and HAP1 (RRID: CVCL_Y019) cells were obtained from Horizon Discovery (Cambridge, UK). HEK293T cells were cultured in Dulbecco's modified Eagle's medium (DMEM; Gibco, Waltham, MA, USA) supplemented with 10% fetal bovine serum (FBS; Gibco), 0.5% nonessential amino acids, 1% penicillin/streptomycin solution at 37 °C with 5% CO_2_. For cell density–dependent assays, cells were seeded at low cell density (1.3 × 10^2^ cells·mm^−2^) or high cell density (2.6 × 10^3^ cells·mm^−2^). For transfection, Lipofectamine 3000 (Invitrogen, Carlsbad, CA, USA) was used according to the manufacturer's instructions. For proteasome inhibition, cells were treated with 1 μm MG132 for 24 h, with DMSO‐treated cells as controls. For nuclear export inhibition, HDC cells were incubated with 20 nm Leptomycin B (LMB; LC Laboratories, Woburn, MA, USA) for 3 h. All cell lines were authenticated within the past 3 years by short tandem repeat (STR) profiling. All cell lines were routinely tested and confirmed to be mycoplasma‐free.

### Reverse transcription‐quantitative PCR (RT‐qPCR)

Total RNA was isolated from wild‐type (WT) and SETDB1 knockout (KO) cells using the RNeasy Plus Mini Kit (Qiagen, Hilden, Germany) according to the manufacturer's instructions. Reverse transcription was performed with 2 μg of total RNA using random hexamers and SuperScript III Reverse Transcriptase (Thermo, Waltham, MA, USA). Quantitative real‐time PCR (qPCR) was conducted using SYBR Green PCR Master Mix (Applied Biosystems, Foster City, CA, USA) on a QuantStudio 3 system. Gene expression levels were normalized to *GAPDH* using the ΔΔ*C*_t_ method to determine fold changes. Primer sequences are listed in the Table S2.

### Immunofluorescence

Cells on poly‐l‐lysine (Sigma, St. Louis, MO, USA)‐coated coverslips were fixed in 4% formaldehyde and permeabilized with permeabilization buffer. After blocking with blocking buffer, cells were incubated with primary antibodies overnight at 4 °C and with Alexa Fluor 488‐ or 594‐conjugated secondary antibodies for 1 h at RT. Nuclei were counterstained with DAPI (Vectashield, Neward, CA, USA), and samples were mounted and imaged using a Zeiss LSM800 confocal microscope (Carl Zeiss, Jena, Germany). Staining was performed in at least three independent experiments, and images were acquired and analyzed with the zen software (Carl Zeiss).

### Subcellular fractionation

Subcellular fractionation was performed using the Nuclear Extraction Kit (Active Motif, Carlsbad, CA, USA). Briefly, cells were resuspended in Hypotonic Buffer and centrifuged to collect the cytoplasmic fraction. The pellet was subsequently extracted with Complete Lysis Buffer, and the supernatant obtained after centrifugation was collected as the nuclear fraction.

### Immunoprecipitation (IP) and chromatin‐IP (ChIP) assays

Cells were harvested 24 h post‐transfection and lysed in lysis buffer. Clarified lysates were incubated overnight at 4 °C with antibody‐bound Dynabeads (Invitrogen), washed, and eluted in SDS loading buffer for immunoblotting. For ChIP, cells were cross‐linked with 0.75% formaldehyde, quenched with glycine, and lysed. Chromatin was sonicated to 200–1000 bp fragments, diluted in RIPA buffer, and incubated with antibodies and pre‐blocked Protein A/G beads (Thermo). After sequential washes (low‐salt, high‐salt, LiCl), complexes were eluted, reverse cross‐linked, and DNA was purified for qPCR. Re‐ChIP was performed by subjecting the first eluate to a second IP with reciprocal antibodies (SETDB1 and FLAG‐TEAD1) under identical wash and elution conditions. Primer sequences and buffer compositions are listed in Tables S3 and S4.

### Western blot analysis

For western blot analysis, cells were harvested and lysed in lysis buffer at 4 °C for 1 h, followed by centrifugation at 13 000 ***g*** for 20 min. Protein concentrations were measured using Bradford reagent (Bio‐Rad, Hercules, CA, USA), and the lysates were boiled in SDS/PAGE loading buffer (Biosesang, Yongin, Republic of Korea) at 95 °C for 10 min. The denatured samples were then electrophoresed on an SDS/PAGE gel and transferred onto a PVDF membrane (Bio‐Rad). After transfer, the membrane was blocked, followed by overnight incubation at 4 °C with the primary antibody. The membrane was then washed three times and incubated with the appropriate HRP‐conjugated secondary antibody. After an additional three washes, the signal was detected using a chemiluminescent substrate (Amersham, Little Chalfont, UK). Antibodies used for both immunofluorescence and western blotting are listed in Table S5.

### YAP1–TEAD target gene analysis

For the MDA‐MB‐231 cells, YAP1 and TEAD4 ChIP‐seq peaks (GSE66081, narrowPeak) were intersected with bedtools and annotated to promoters (±1.5 kb from the TSS) using gencode v19, thereby defining promoter‐proximal binding sites. For HEK293 cells, YAP1, TEAD1, and TEAD4 ChIP‐seq signal tracks (GSE130135, bigWig) were processed with a signal cutoff (≥ 15) converted to BED, and used to identify shared peaks (YAP1–TEAD1/4) with *pyRanges*, which were similarly annotated to promoters. Resulting YAP1–TEAD target lists were compared with DEGs from RNA‐seq of three SKO HEK293T clones from our previous study [21]. Genes present in both datasets were defined as SETDB1‐regulated YAP1–TEAD targets.

## Results

### SETDB1 displays cell density‐dependent nucleocytoplasmic movement along with ATF7IP

**Fig. 1:**
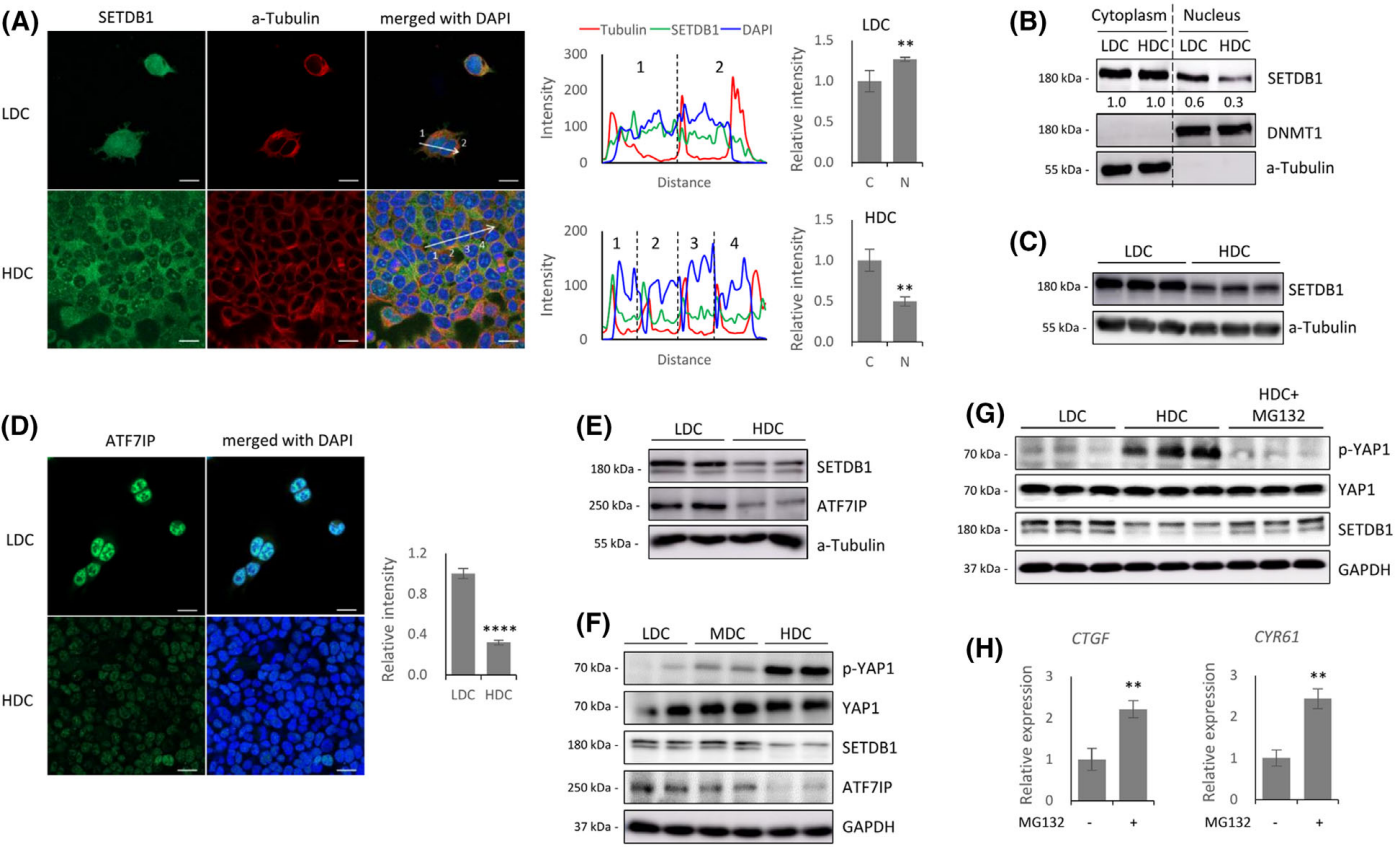
SETDB1 undergoes cell density–dependent nuclear exclusion in coordination with ATF7IP. (A) Immunofluorescence staining of SETDB1 (green), α‐Tubulin (red), and DAPI (blue) in HEK293T cells under low (LDC) and high (HDC) cell‐density culture. SETDB1 is distributed throughout the cytoplasm and nucleus under LDC, but predominantly cytoplasmic under HDC. Line‐scan fluorescence intensity profiles (middle) and quantification of nuclear‐to‐cytoplasmic intensity ratios (right) confirm a significant reduction in nuclear SETDB1 signal under HDC. (B) Subcellular fractionation of LDC and HDC HEK293T cells. Western blot analysis shows decreased nuclear SETDB1 under HDC. DNMT1 and α‐Tubulin were used as nuclear and cytoplasmic fraction controls, respectively. (C) Reduction in total SETDB1 protein levels under HDC. (D) Immunofluorescence (IF) staining. ATF7IP shows strong nuclear localization under LDC, which is significantly reduced under HDC. Quantification of nuclear ATF7IP intensity is shown on the right. (E) Reduction in total SETDB1 and ATF7IP protein levels under HDC. (F) Western blot across increasing cell densities (LDC, MDC, HDC). A gradual loss of SETDB1 and ATF7IP protein levels, along with a progressive increase in p‐YAP1, is shown. (G) MG132 treatment. It restores total SETDB1 levels under HDC and concurrently reduces p‐YAP1 levels. (H) Quantitative real‐time PCR (qPCR) analysis showed increased expression of YAP1–TEAD target genes *CTGF* and *CYR61* following MG132 treatment under HDC conditions. Statistical significance was determined by unpaired two‐tailed Student's *t*‐test; data are presented as mean ± SD from at least three independent experiments; **P < 0.01, ****P < 0.0001. Scale bars: 20 μm.

We found that SETDB1 localization is regulated in a cell density‐dependent manner in HEK293T cells. Confocal microscopy revealed that SETDB1 was evenly distributed between the nucleus and cytoplasm under low‐density culture (LDC), but became predominantly cytoplasmic under high‐density culture (HDC), indicating a loss of nuclear SETDB1 (Fig. 1A). This nuclear reduction was confirmed by subcellular fractionation (Fig. 1B and Fig. S1A). In parallel, we observed a substantial decrease in total SETDB1 protein levels under HDC (Fig. 1C). Notably, *SETDB1* transcript levels remained unchanged between LDC and HDC (Fig. S1B), suggesting a regulation at the protein level. A similar density‐dependent movement pattern was observed in human near‐haploid (HAP1) cells, where immunostaining and western blotting both revealed reduced nuclear and total SETDB1 levels in HDC (Fig. S1C,D).

Given that SETDB1 and ATF7IP are known to stabilize each other in the nucleus [18, 20, 22], we examined whether SETDB1 dynamics were linked to ATF7IP. Immunostaining showed a sharp decrease in nuclear ATF7IP under HDC (Fig. 1D), and western blotting confirmed reductions in both SETDB1 and ATF7IP proteins (Fig. 1E). A similar result was obtained in HAP1 cells (Fig. S1E). *ATF7IP* transcript levels were unaffected by cell density (Fig. S1F), indicating a protein‐level regulation. This cell density‐dependent behavior of ATF7IP aligns well with, and thus supports, the distinct nuclear‐to‐cytoplasmic distribution of SETDB1 between LDC and HDC.

### Proteasomal degradation of SETDB1 explains its nuclear depletion in HDC

The cell density‐dependent distribution of SETDB1 closely parallels that of YAP1, the primary effector of the Hippo signaling pathway [17]. Consistent with this, we observed that YAP1, which is predominantly nuclear under LDC, becomes largely cytoplasmic under HDC in HEK293T cells (Fig. S1G). This is in line with Hippo pathway activation in HDC, which promotes phosphorylation of YAP1 at Ser127, leading to its nuclear exclusion [23]. Correspondingly, we detected a gradual increase in phosphorylated YAP1 (p‐YAP1) levels as cell density increased, while total YAP1 protein levels remained stable (Fig. 1F).

To determine whether proteasomal degradation contributes to the loss of nuclear SETDB1 under HDC, we treated HEK293T cells with MG132, a proteasome inhibitor that blocks the ubiquitin–proteasome pathway [24]. MG132 treatment significantly restored total SETDB1 protein levels in HDC, nearly reaching those observed in LDC (Fig. 1G). In parallel, immunostaining showed a clear enrichment of nuclear SETDB1 signals following MG132 treatment, indicating that proteasome‐mediated degradation is a major cause of SETDB1 loss from the nucleus at high density (Fig. S2A,B). Meanwhile, expression levels of YAP1‐TEAD target genes (YTGs) were accordingly increased (Fig. 1H, see Discussion).

### SETDB1 enhances YAP1 phosphorylation

**Fig. 2:**
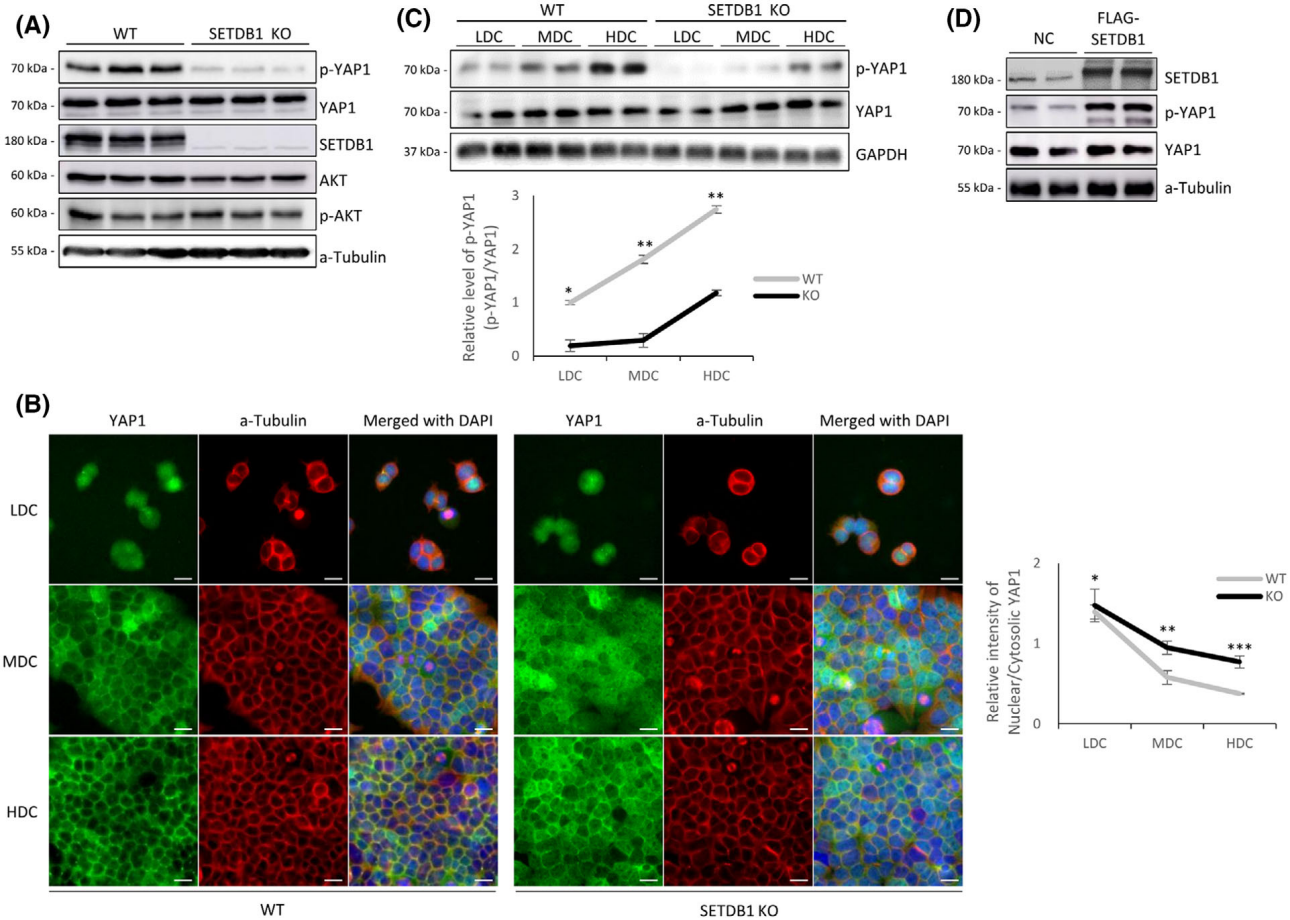
SETDB1 promotes YAP1 phosphorylation and restricts its nuclear localization in a cell density‐dependent manner. (A) Western blot analysis of YAP1, AKT, and SETDB1 in HEK293T wild‐type (WT) and SETDB1 knockout (KO) cells. p‐YAP1 (S127) was markedly reduced in KO cells, while total YAP1, AKT, and p‐AKT (T308) levels remain unchanged. (B) Immunofluorescence staining of YAP1 (green) in WT and SETDB1 KO cells under low (LDC), medium (MDC), and high (HDC) cell‐density culture. In WT cells, YAP1 translocates from the nucleus to the cytoplasm as cell density increases, whereas KO cells retain nuclear YAP1. On the right, quantification of nuclear‐to‐cytoplasmic YAP1 intensity ratios shows significantly enhanced nuclear YAP1 localization in KO cells. (C) Western blot of p‐YAP1 (S127) and total YAP1 levels across increasing cell‐density culture conditions (LDC, MDC, HDC) in WT and KO cells. Densitometric quantification of p‐YAP1/YAP1 ratios confirms reduced phosphorylation in KO cells at all densities. Total YAP1 blot in Fig. 1F is reused here, as both derive from the same experiment using lysates prepared simultaneously from the same cells. (D) Transient overexpression of Flag‐SETDB1 in HEK293T cells. SETDB1 overexpression increases p‐YAP1 levels without altering total YAP1. Statistical significance was determined by unpaired two‐tailed Student's *t*‐test; data are presented as mean ± SD from at least three independent experiments; *P < 0.05, **P < 0.01, ***P < 0.001, n.s., not significant. Scale bars: 20 μm.

To investigate whether SETDB1 modulates YAP1 activity, we performed western blotting in three SETDB1 knockout (SKO) HEK293T clones [21]. While total YAP1 levels remained unchanged, p‐YAP1 levels were markedly reduced under HDC in SKO cells (Fig. 2A), suggesting that SETDB1 promotes YAP1 phosphorylation. Immunostaining confirmed abnormal nuclear accumulation of YAP1 in SKO cells under HDC (Fig. 2B), likely reflecting its unphosphorylated form. Particularly notable was the medium‐density culture (MDC), where YAP1 in SETDB1‐intact cells began to relocate from the nucleus to the cytoplasm and prominently accumulated at cell–cell junctions along the plasma membrane. In contrast, in SKO cells, YAP1 remained diffusely distributed with little membrane localization.

Because SETDB1 loss in HDC coincides with a reduction in nuclear SETDB1 levels, we tested whether restoring the nuclear pool of SETDB1 would rescue YAP1 phosphorylation. Treatment of HDC cells with the nuclear export inhibitor leptomycin B (LMB) effectively retained SETDB1 in the nucleus without altering its overall abundance (Fig. S3A,B). However, LMB treatment did not increase YAP1 Ser127 phosphorylation (Fig. S3B), indicating that nuclear retention of SETDB1 alone is insufficient to restore Hippo pathway activity.

Analysis across cell densities further showed that YAP1 phosphorylation still occurred without SETDB1 but was substantially less efficient (Fig. 2C), suggesting that SETDB1 facilitates, but is not strictly required for, YAP1 phosphorylation. Co‐immunoprecipitation assays did not detect direct interactions between SETDB1 and the Hippo kinases MST1 or LATS1 (data not shown), suggesting that SETDB1 does not act through direct physical association with core Hippo components. Because SETDB1 has been reported to activate AKT [9, 25] and AKT can influence YAP1 phosphorylation [26], we examined AKT status; however, total and phosphorylated AKT levels were unchanged in SKO cells (Fig. 2A), excluding AKT involvement. Consistent with an indirect mechanism, overexpression of SETDB1 increased p‐YAP1 levels (Fig. 2D). Together, these findings indicate that SETDB1 enhances YAP1 phosphorylation through a noncanonical pathway independent of nuclear localization, AKT signaling, and direct interaction with core Hippo kinases.

### SETDB1 represses YAP1–TEAD target gene expression

**Fig. 3:**
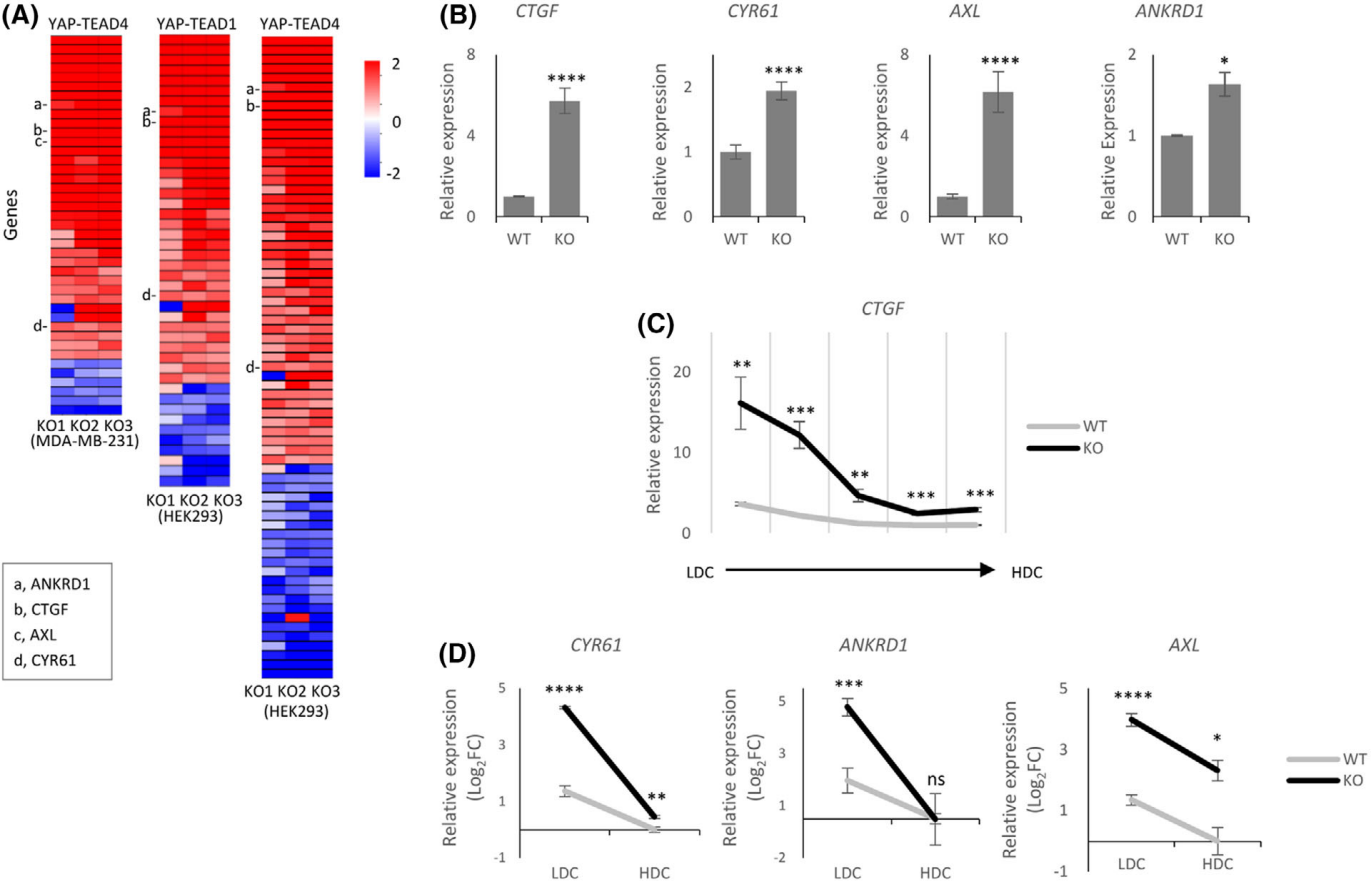
SETDB1 represses YAP1‐TEAD target gene (YTG) expression. (A) Heatmap of differentially expressed YAP1–TEAD target genes (YTGs) (*P* < 0.05, |log_2_FC| > 1) from RNA‐seq analysis of three independent SETDB1‐knockout HEK293T clones compared to wild‐type (WT) cells. Color scale represents log_2_ fold change. YTGs selected for further validation in this study (*CTGF*, *CYR61*, *ANKRD1*, *AXL*) are indicated. (B) qPCR validation of representative YTGs (*CTGF*, *CYR61*, *AXL*, *ANKRD1*), showing significant upregulation in KO cells relative to WT. (C) *CTGF* expression across increasing cell‐density culture conditions (LDC to HDC). Expression is elevated in KO cells compared to WT, particularly under LDC. (D) qPCR analysis of additional YTGs (*CYR61*, *ANKRD1*, *AXL*) under LDC and HDC. YTG upregulation in KO cells is prominent under LDC but diminishes in HDC. Data are shown on a log_2_ scale. Statistical significance was determined by unpaired two‐tailed Student's *t*‐test; data are presented as mean ± SD from at least three independent experiments; *P < 0.05, **P < 0.01, ***P < 0.001, ****P < 0.0001; n.s., not significant.

Since it is well‐established that phosphorylation of YAP1 leads to its nuclear exclusion and loss of transcriptional activity on YTGs [17], our results suggest that YTG expressions should be upregulated in the absence of SETDB1. We analyzed the transcriptomes of three different SKO clones using RNA sequencing (RNA‐seq) data from our previous study [21]. To define YTGs, we first curated a reference list of YAP1–TEAD1 or YAP1–TEAD4 common target genes by retrieving promoter‐associated peaks (TSS ± 1.5 kb) from public YAP1‐TEAD1/4 ChIP‐seq datasets from the MDA‐MB‐231 breast cancer cell line (GSE66081; [27]) and HEK293 cells (GSE130135; [28]). These YTGs were then intersected with differentially expressed genes identified between WT and SKO cells (*P* < 0.05, |log_2_ fold change| > 1) to identify YTGs that are differentially expressed in SKO cells. As expected, most differentially expressed YTGs were upregulated in SKO cells (Fig. 3A and Tables S6–S8). These results suggest that SETDB1 plays a broad role in repressing YTG expression. qPCR validation confirmed increased expression of canonical YTGs such as *CTGF*, *CYR61*, *AXL*, and *ANKRD1* (Fig. 3B, and see also Fig. 3A).

To further investigate how SETDB1 regulates YTG expression, we examined *CTGF* expression across different cell densities. Under LDC conditions, *CTGF* expression was markedly elevated in SKO cells compared to controls (Fig. 3C), suggesting that SETDB1 potently represses *CTGF* transcription when both SETDB1 and YAP1 are nuclear. As cell density increased, *CTGF* levels declined in SKO cells and approached those of control cells in HDC. A similar trend was observed for other YTGs (Fig. 3D). These results indicate that SETDB1 contributes to YTG repression even under LDC conditions, where YAP1 is nuclear and transcriptionally active. Although YTG expression is relatively high in LDC, the further elevation observed in SKO cells suggests that SETDB1‐mediated repression is functionally operative.

On the other hand, as mentioned above (Fig. 1G,H), MG132 treatment under HDC conditions led to an increase in SETDB1 protein levels, a reduction in p‐YAP1, and a concomitant upregulation of YTG expression. MG132 treatment has also been reported to reduce YAP1 phosphorylation and increase expression of YTG [29, 30]. Although SETDB1 levels were restored, the simultaneous induction of YTGs suggests that this SETDB1 is not functionally active in repressing transcription. Instead, the reduction in p‐YAP1 likely accounts for YAP1 activation and YTG upregulation. Together with the results in Fig. 3C, these findings suggest that MG132‐stabilized SETDB1 may either be retained in the cytoplasm or persist in the nucleus in a transcriptionally inactive, possibly ubiquitinated state.

### SETDB1 competes with YAP1 for TEAD1 binding and represses YAP1‐TEAD target genes

**Fig. 4:**
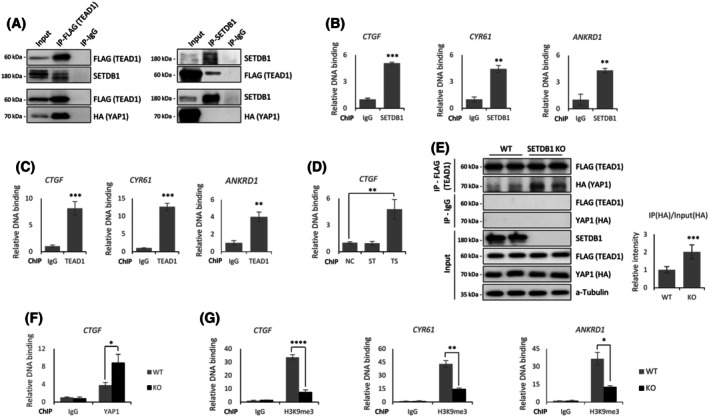
SETDB1 interacts with TEAD1 and competes with YAP1 for TEAD1 binding to repress target gene transcription. (A) Co‐immunoprecipitation (co‐IP) analysis in HEK293T cells co‐expressing GFP‐SETDB1, FLAG‐TEAD1, and HA‐YAP1. FLAG pull‐down (left) confirms interactions between TEAD1 and both YAP1 and SETDB1. SETDB1 pull‐down (right) detects interaction with TEAD1, but not with YAP1. (B, C) Chromatin IP (ChIP)‐qPCR analysis of *CTGF*, *CYR61*, and *ANKRD1* promoter occupancy by SETDB1 (B) and Flag‐tagged TEAD1 (C). IgG served as a negative control. (D) Sequential ChIP (Re‐ChIP) assay showing that TEAD1 → SETDB1 (TS) pull‐down results in *CTGF* promoter enrichment, while the reverse order (ST) does not. NC, negative control. (E) Co‐IP using lysates from wild‐type (WT) and SETDB1‐KO HEK293T cells shows enhanced YAP1–TEAD1 interaction in the absence of SETDB1. Quantification from three independent experiments (right panel), normalized to input levels, reveals an approximately two‐fold increase in YAP1‐TEAD1 binding in SETDB1‐KO cells. (F, G) ChIP‐qPCR analysis showing increased YAP1 occupancy at the *CTGF* promoter (F) and decreased H3K9me3 enrichment at the *CTGF*, *CYR61*, and *ANKRD1* promoters (G) in SETDB1 KO cells compared to WT. Statistical significance was determined by unpaired two‐tailed Student's *t*‐test; data are presented as mean ± SD from at least three independent experiments; *P < 0.05, **P < 0.01, ***P < 0.001, ****P < 0.0001; n.s., not significant.

Beyond its role in modulating YAP1 phosphorylation, we next investigated whether SETDB1 also represses YTG transcription at the chromatin level. Specifically, we tested whether SETDB1 physically interacts with Hippo pathway downstream transcriptional effectors, such as YAP1 and TEAD1. Co‐IP confirmed the known interaction between YAP1 and TEAD1 [23], but revealed no detectable interaction between SETDB1 and YAP1. Instead, SETDB1 directly interacted with TEAD1 (Fig. 4A). Chromatin‐IP (ChIP) followed by qPCR confirmed that both SETDB1 and TEAD1 were enriched at the *CTGF*, *CYR61* and *ANKRD1* promoters (Fig. 4B,C). Sequential ChIP (re‐ChIP) analysis further revealed enrichment only in the TEAD1 → SETDB1 direction, suggesting that SETDB1 is recruited by TEAD1 (Fig. 4D).

To determine the functional consequences of this interaction, we examined YAP1–TEAD1 binding in SKO cells. Co‐IP revealed increased YAP1–TEAD1 association in the absence of SETDB1 (Fig. 4E), suggesting that SETDB1 competes with YAP1 for TEAD1 binding. Consistently, ChIP‐qPCR showed increased YAP1 occupancy at the *CTGF* promoter in SKO cells (Fig. 4F). In addition to this competition‐based mechanism, we next examined whether SETDB1 also represses transcription through its enzymatic activity. ChIP‐qPCR analysis of the *CTGF*, *CYR61*, and *ANKRD1* promoters revealed significantly reduced H3K9me3 enrichment in SKO cells (Fig. 4G), indicating that SETDB1 promotes a repressive chromatin state at YTG loci via its histone methyltransferase activity. Together, these findings suggest that SETDB1 represses YTG transcription through a dual mechanism: by competing with YAP1 for TEAD1 binding at target loci, and by depositing H3K9me3 to establish a repressive chromatin environment. Through this combined mode of action, SETDB1 effectively antagonizes YAP1‐TEAD‐mediated activation of Hippo target genes.

## Discussion

Our findings demonstrate that SETDB1 undergoes dynamic subcellular localization in a cell density‐dependent manner. In LDC, SETDB1 accumulates in the nucleus, whereas in HDC, it is excluded from the nucleus and subsequently degraded via the proteasome pathway. This SETDB1 redistribution appears to be post‐translationally regulated and involves ATF7IP, a known interactor and stabilizer of SETDB1, which is similarly downregulated at the protein level in HDC. Although the exact mechanism behind the cell density‐dependent regulation of SETDB1 remains unclear, one plausible explanation involves the E3 ubiquitin ligase Von Hippel–Lindau (VHL). VHL has been reported to interact with SETDB1 in HEK293T cells, promoting its ubiquitination and proteasomal degradation, which can be inhibited by bortezomib [31]. Consistently, in our study, MG132—a functional analog of bortezomib—restored SETDB1 protein levels in HDC (Fig. 1G), suggesting degradation via the ubiquitin‐proteasome system [32]. Notably, VHL itself exhibits cell density‐dependent behavior: It is nuclear and unstable in LDC but becomes stabilized and accumulates in the cytoplasm in HDC, where its E3 ligase activity increases [33, 34, 35]. This aligns with our observation that SETDB1 level diminished from the nucleus and degraded in HDC, raising the possibility that VHL mediates SETDB1 degradation under HDC conditions. However, we did not directly assess VHL expression or localization under our experimental conditions to verify this hypothesis. While the behaviors of VHL and SETDB1 appear to be correlated, direct evidence for VHL‐driven nuclear export of SETDB1 is lacking, and this hypothesis remains speculative.

Although MG132 treatment restored nuclear SETDB1 levels at high cell density, YAP1–TEAD target genes remained upregulated, creating an apparent paradox. Our data, together with prior studies, suggest two mechanisms that may account for this observation. First, proteasome inhibition prevents SETDB1 degradation but does not necessarily restore its catalytic function; the protein may remain polyubiquitinated, a modification known to impair the activity of several chromatin regulators under MG132 treatment [36, 37, 38]. Second, MG132 exerts broad effects on Hippo signaling independently of SETDB1. Specifically, MG132 disrupts the Hsp70–BAG3–AMOTL2 complex, thereby impairing LATS1‐mediated phosphorylation of YAP1 at Ser127 and facilitating nuclear YAP1 accumulation, ultimately enhancing YAP1–TEAD transcriptional output [29, 30]. Consistent with this mechanism, we observed a reduction in phosphorylated YAP1 (S127) following MG132 treatment. Thus, the increase in YAP1–TEAD activity despite restored nuclear SETDB1 most likely reflects a combination of reduced SETDB1 functionality and MG132‐induced activation of upstream Hippo regulatory pathways.

We showed that SETDB1 modulates the Hippo signaling pathway, particularly by regulating YAP1 phosphorylation. SETDB1 depletion led to a marked reduction in phosphorylated YAP1, resulting in the nuclear accumulation of its active form. Consequently, YTG expression fails to be turned off, leading to delayed transcriptional repression. These findings suggest that SETDB1 contributes to Hippo pathway output not only through chromatin‐level repression of YAP1–TEAD1 target genes but also by maintaining the upstream phosphorylation‐dependent inactivation of YAP1. Although the precise mechanism remains unclear, SETDB1 may influence the localization or activity of upstream kinases such as MST1 and LATS1/2, or stabilize components required for YAP1 phosphorylation [23]. While these canonical Hippo kinases are essential for YAP1 phosphorylation, our IP experiments failed to detect a physical interaction between SETDB1 and these kinases. This raises the possibility that SETDB1 regulates YAP1 phosphorylation through indirect or context‐dependent mechanisms—potentially involving chromatin‐associated scaffolds or alternative signaling intermediates such as HDAC6‐dependent RhoA–ROCK signaling, which is impaired upon SETDB1 loss [11, 39], or SETDB1‐promoted p38 and AKT signaling [40, 41, 42]. Nonetheless, the absence of detectable interactions should be interpreted with caution, as such results may reflect technical limitations or the inherently transient nature of weak protein–protein associations that are difficult to capture via standard IP‐based methods. Moreover, LMB‐mediated nuclear retention of SETDB1 in HDC did not increase YAP1 phosphorylation (Fig. S3B), indicating that nuclear localization alone is insufficient for Hippo pathway activation.

Beyond its upstream effects, SETDB1 also acts directly at the chromatin level to suppress Hippo pathway output. We found that SETDB1 physically interacts with TEAD1, the DNA‐binding partner of YAP1, but not with YAP1 itself. This interaction enables SETDB1 to localize to YTG promoters—such as *CTGF*—where it co‐occupies chromatin with TEAD1 (Fig. 4). The absence of SETDB1 led to increased YAP1–TEAD1 complex formation and elevated YAP1 occupancy at these promoters, suggesting that SETDB1 competes with YAP1 for TEAD1 binding. Moreover, as a H3K9 methyltransferase, SETDB1 deposits repressive H3K9me3 marks at these loci, contributing to local, facultative heterochromatin formation and gene silencing. Thus, SETDB1 not only limits YAP1 activity upstream via phosphorylation but also downstream by restricting YAP1‐driven transcription at the chromatin level. These dual actions significantly expand our understanding of how epigenetic regulators such as SETDB1 can modulate signal‐dependent gene expression.

**Fig. 5:**
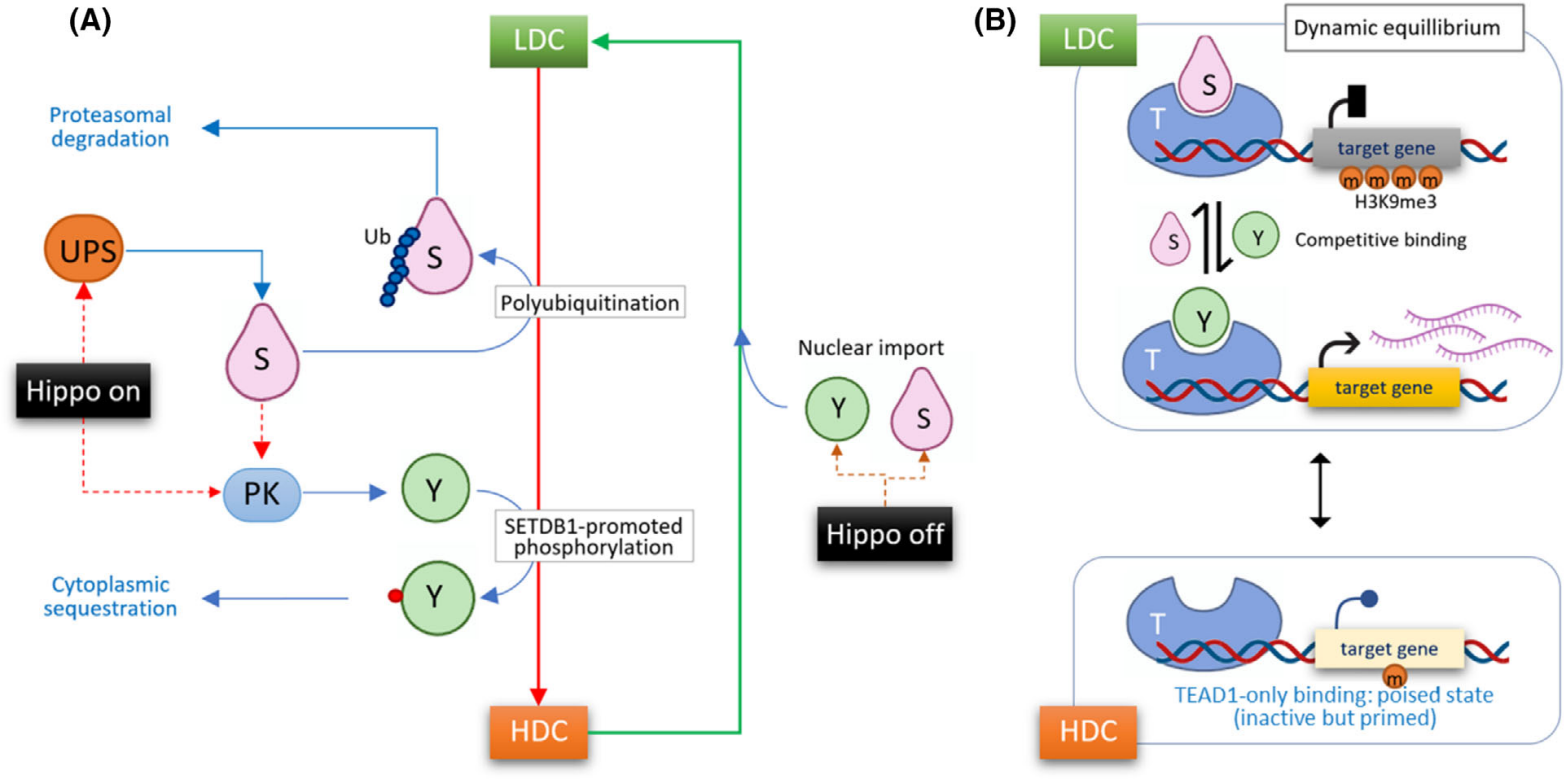
SETDB1 controls Hippo pathway output and YAP1‐TEAD1 transcriptional equilibrium through dual mechanisms. (A) Cell density–dependent regulation of SETDB1 and YAP1. Under low cell‐density culture (LDC) conditions, the Hippo pathway is inactive (“Hippo off”), allowing both YAP1 and SETDB1 to translocate into the nucleus. In contrast, under high cell‐density culture (HDC), the Hippo pathway is activated (“Hippo on”), leading to two major outcomes: (a) YAP1 is phosphorylated by protein kinases (PKs) and sequestered in the cytoplasm; (b) SETDB1 undergoes polyubiquitination and proteasomal degradation via the ubiquitin–proteasome system (UPS). Notably, SETDB1 also promotes YAP1 phosphorylation during LDC‐to‐HDC transition, suggesting that SETDB1 facilitates Hippo pathway activation. While SETDB1 appears to act upstream of YAP1 phosphorylation, the exact identity of the kinase(s) involved and the molecular mechanism of SETDB1's influence on PK activity remain to be elucidated. (B) TEAD1‐centric transcriptional control mediated by competitive binding of YAP1 and SETDB1. In the nucleus, TEAD1 serves as a central platform for transcriptional regulation. Under LDC conditions, nuclear YAP1 and SETDB1 competitively bind to TEAD1. YAP1 promotes transcription of TEAD1 target genes, whereas SETDB1 deposits H3K9me3 at their promoters to establish repressive chromatin. This dynamic equilibrium determines whether target genes are activated or silenced. Under HDC conditions, both YAP1 and SETDB1 are excluded from the nucleus, and TEAD1 remains bound to chromatin without co‐activators or co‐repressors. This TEAD1‐only state may represent a transcriptionally poised configuration, potentially maintaining the locus in a state that is inactive but responsive to upstream cues.

Figure 5 illustrates a model in which cell density–dependent SETDB1 shuttling coordinates with Hippo pathway activity to regulate YTG transcription. Under LDC, Hippo signaling is inactive, allowing both YAP1 and SETDB1 to stay in the nucleus. In this context, TEAD1 at Hippo target gene promoters serves as a binding platform for either YAP1 or SETDB1. These two factors compete for TEAD1 binding, establishing a YAP1‐biased dynamic equilibrium: SETDB1 binding leads to transcriptional repression via H3K9me3 deposition, whereas YAP1 binding promotes gene activation. As cells proliferate and reach HDC, Hippo signaling is turned on. Activated protein kinases phosphorylate YAP1, triggering its nuclear exit. Concurrently, SETDB1 is targeted for ubiquitination by the ubiquitin–proteasome system (UPS), resulting in its cytoplasmic translocation and degradation. These processes collectively clear both YAP1 and SETDB1 from the nucleus, thereby disrupting their transcriptional control over Hippo target genes. SETDB1 thus functions as a context‐dependent transcriptional repressor whose nuclear availability is dynamically restricted by Hippo pathway activation.

YAP1 signaling is known to respond rapidly to changes in mechanical and spatial cues, such as cell density, matrix stiffness, and cell–cell contact [43]. This dynamic responsiveness enables cells to quickly adjust transcriptional programs during tissue growth, regeneration, or contact inhibition. Therefore, once Hippo signaling is activated, the prompt inactivation of YAP1 must be coupled with efficient transcriptional repression of its target genes to prevent inappropriate or residual gene activation. Our model suggests that SETDB1 fulfills this role by acting as a transcriptional safeguard. Although YAP1 is excluded from the nucleus upon Hippo activation, TEAD1 remains chromatin‐bound and may sustain a transcriptionally permissive, poised state [44]. In this context, SETDB1‐mediated H3K9me3 deposition may rapidly convert this state into a repressed chromatin configuration, thereby enforcing the timely shut‐off of Hippo target genes.

While these insights highlight a novel dual role for SETDB1 in regulating Hippo signaling, several limitations should be considered. Most of our mechanistic conclusions are based on a single cell line model (HEK293T) under *in vitro* conditions, which may not fully capture the complexity of Hippo regulation in other cellular contexts. Additional studies using diverse cell types, primary tissues, and *in vivo* models (e.g., mouse genetics or organoids) are needed to validate the broader relevance of our findings. Identifying SETDB1‐dependent regulators of Hippo kinases—or upstream repressors of YAP1—will be key to clarifying this relationship. It will also be of interest to explore whether SETDB1 intersects with other mechanotransduction pathways, such as Wnt/β‐catenin or TGF‐β signaling, which similarly respond to mechanical cues such as cell density and matrix stiffness [14, 15].

Together, our findings establish SETDB1 as a dual‐function regulator that integrates extracellular cues with intracellular signaling and epigenetic control. By dynamically shuttling in response to cell density, SETDB1 coordinates with the Hippo pathway to regulate both the phosphorylation‐dependent inactivation and the chromatin‐level repression of YAP1. This dual mechanism not only ensures the timely silencing of Hippo target genes but also highlights SETDB1 as a key node linking mechanical inputs to transcriptional outcomes. More broadly, our study expands the functional landscape of histone methyltransferases beyond static gene silencing and positions SETDB1 as a context‐sensitive effector of signal‐dependent transcriptional regulation.

## Author contributions

JE was involved in writing—original draft, investigation, formal analysis, data curation, conceptualization. JJ was involved in investigation, validation. JSP was involved in investigation, validation. YKK was involved in writing—review and editing, supervision, project administration, funding acquisition, conceptualization.

## Supporting information


**Fig. S1.** Cell density–dependent regulation of SETDB1 and ATF7IP is conserved in HAP1 cells.
**Fig. S2.** MG132 restores nuclear SETDB1 at high cell‐density culture.
**Fig. S3.** Leptomycin B (LMB) retains SETDB1 in the nucleus but does not restore its stability or enhance YAP1 phosphorylation.

**Table S1.** Primers used in the vector construction.
**Table S2.** RT‐qPCR primers.
**Table S3.** ChIP‐qPCR primers.
**Table S4.** Buffer compositions.
**Table S5.** Antibody information.
**Table S6.** Differentially expressed genes in MDA‐MB‐231 (YAP1‐TEAD4 target genes).
**Table S7.** Differentially expressed genes in HEK293 (YAP1‐TEAD1 target genes).
**Table S8.** Differentially expressed genes in HEK293 (YAP1‐TEAD4 target genes).

## Data Availability

The data that support the findings of this study are available from the corresponding author upon reasonable request.
