# Supplementary material for: Cell density–dependent nuclear‐cytoplasmic shuttling of SETDB1 integrates with Hippo signaling to regulate YAP1‐mediated transcription
Source: FEBS Lett. 2026 Jan 19;600(3):370–82. doi: 10.1002/1873-3468.70286 (PMC12883898; doi:10.1002/1873-3468.70286)
Supplement: Supplementary file 1 — Fig. S1. Cell density–dependent regulation of SETDB1 and ATF7IP is conserved in HAP1 cells. Fig. S2. MG132 restores nuclear SETDB1 at high cell‐density culture. Fig. S3. Leptomycin B (LMB) retains SETDB1 in the nucleus but does not restore its stability or enhance YAP1 phosphorylation. [file FEB2-600-370-s002.pdf]

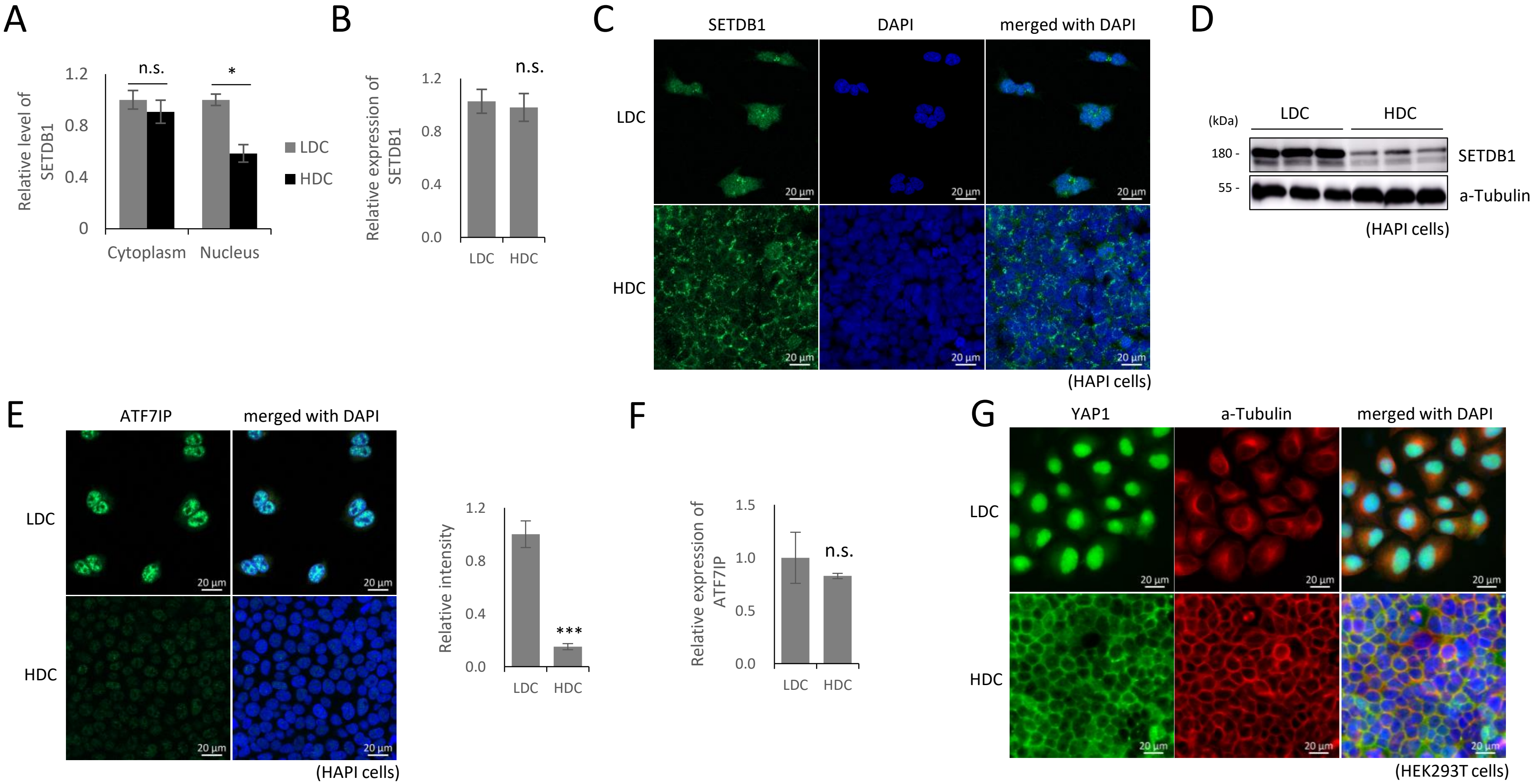

Supplementary Figure S1. Cell density–dependent regulation of SETDB1 and ATF7IP is conserved in HAP1 cells.

(A) Quantification of SETDB1 levels in cytoplasmic and nuclear fractions under LDC and HDC from three independent experiments. SETDB1 levels were normalized to LDC within each compartment.

(B) qPCR analysis of SETDB1 mRNA levels under LDC and HDC conditions shows no significant difference in transcript levels in HEK293T cells.

(C) Immunofluorescence staining of SETDB1 (green) and DAPI (blue) in HAP1 cells. SETDB1 is distributed throughout the cytoplasm and nucleus under LDC, but becomes predominantly cytoplasmic under HDC, similar to HEK293T cells.

(D) Western blot analysis reveals a reduction in total SETDB1 protein levels under HDC in HAP1 cells.

(E) Immunofluorescence staining of ATF7IP (green) and DAPI (blue) in HAP1 cells shows strong nuclear localization under LDC, which is significantly reduced under HDC. Quantification of nuclear ATF7IP intensity is shown on the right.

(F) qPCR analysis shows ATF7IP transcript levels remain unchanged between LDC and HDC in HEK293T cells.

(G) Immunofluorescence staining of YAP1 (green), α-Tubulin (red), and DAPI (blue) in HEK293T cells under LDC and HDC. YAP1 localizes predominantly to the nucleus under LDC, but becomes largely cytoplasmic under HDC.

Statistical significance was determined by unpaired two-tailed Student's t-test; data are presented as mean ± SD from at least three independent experiments; *$p<0.05$, ***$p < 0.001$, n.s., not significant. Scale bars: 20 µm

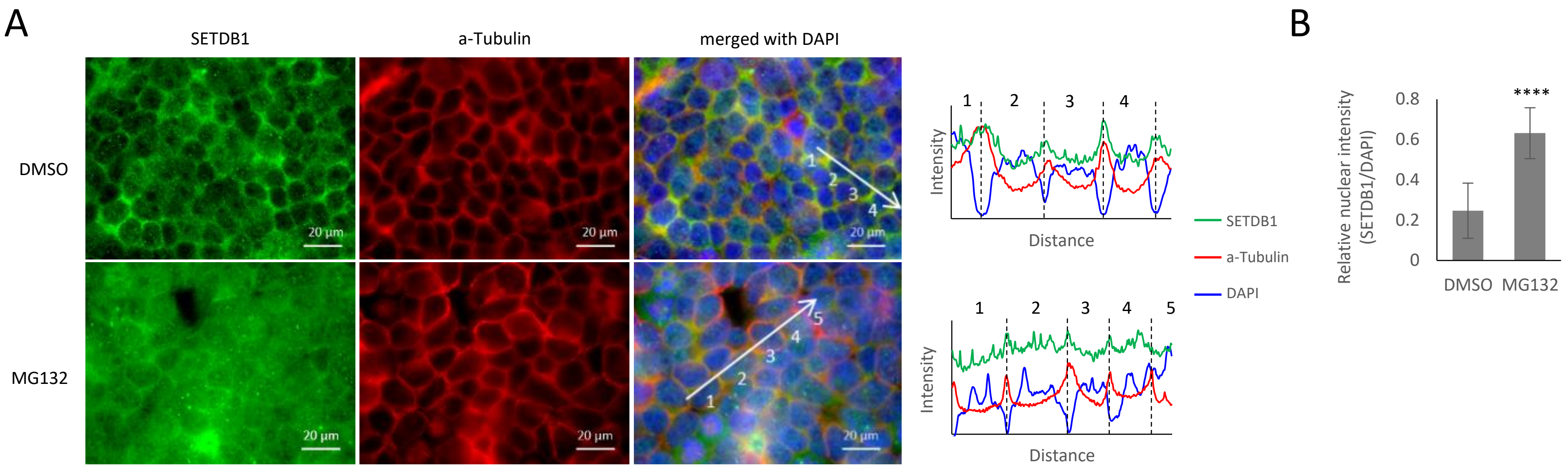

Supplementary Figure S2. MG132 restores nuclear SETDB1 at high cell-density culture.

(A) Immunofluorescence of SETDB1 (green), a-Tubulin (red), and DAPI (blue) in HEK293T cells under high cell-density culture with DMSO or MG132. Line-scan fluorescence intensity profiles (middle) and quantification of nuclear-to-cytoplasmic SETDB1 intensity ratio (right) show restoration of the nuclear pool upon MG132 treatment.

(B) Relative signal intensity of nuclear SETDB1 to DPI in either DMSO (control) or MG132 treated cells. The intensity was measured using the histogram tool in Carl Zeiss Microscopy GMBH.

Statistical significance was determined by unpaired two-tailed Student's t-test; data are presented as mean ± SD from at least three independent experiments; ****$p < 0.0001$. Scale bars: 20 μm

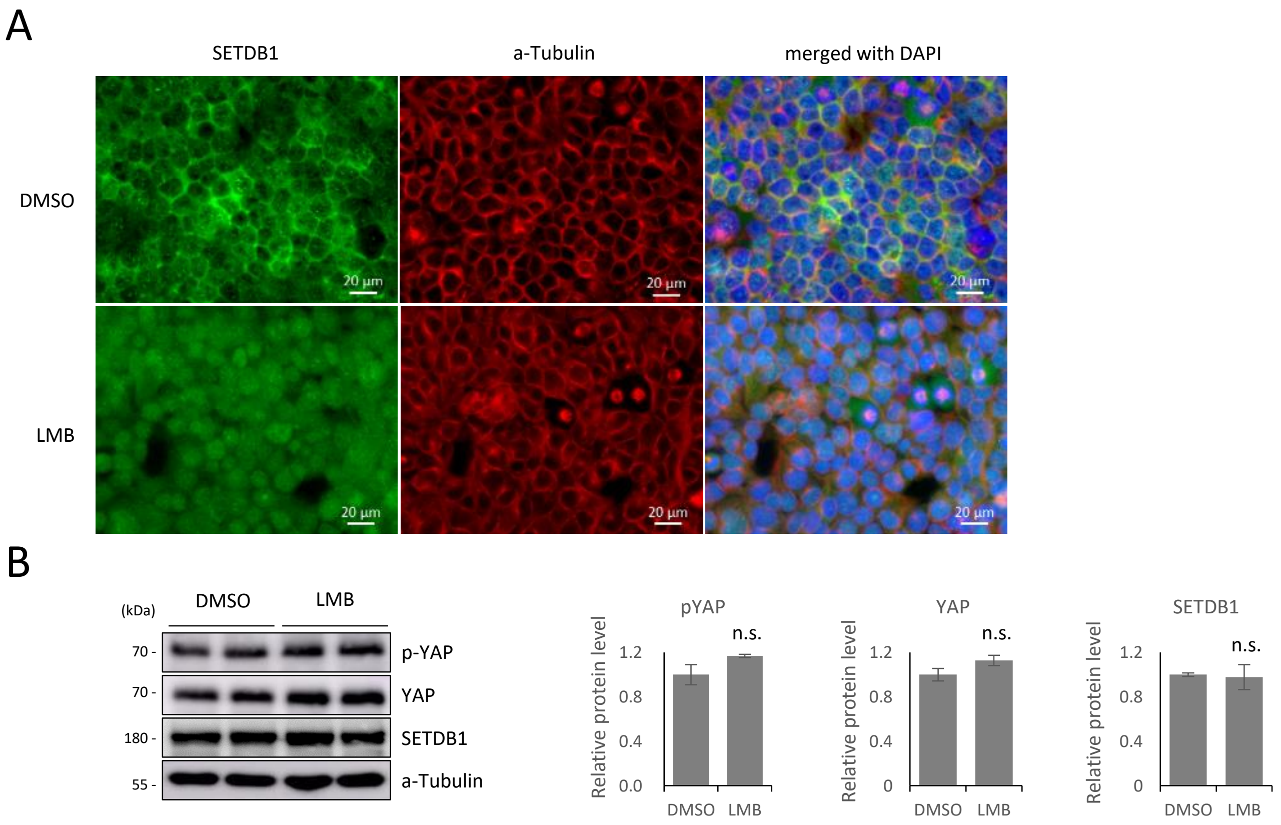

Supplementary Figure S3. Leptomycin B (LMB) retains SETDB1 in the nucleus but does not restore its stability or enhance YAP1 phosphorylation.

(A) Immunofluorescence of SETDB1 (green), a-Tubulin (red), and DAPI (blue) in HEK293T cells under HDC with DMSO or LMB. LMB treatment promotes nuclear retention of SETDB1.

(B) Western blot analyses of YAP1, phospho-YAP1 (Ser127), and SETDB1 under HDC with DMSO or LMB treatment. Quantification shows that LMB treatment does not alter total SETDB1 and YAP1 phosphorylation level.

Statistical significance was determined by unpaired two-tailed Student's t-test; data are presented as mean ± SD from at least three independent experiments; n.s., not-significant. Scale bars: 20 µm
