# Supplementary material for: Cell density–dependent nuclear‐cytoplasmic shuttling of SETDB1 integrates with Hippo signaling to regulate YAP1‐mediated transcription
Source: FEBS Lett. 2026 Jan 19;600(3):370–82. doi: 10.1002/1873-3468.70286 (PMC12883898; doi:10.1002/1873-3468.70286)
Supplement: Supplementary file 2 — Table S1. Primers used in the vector construction. Table S2. RT‐qPCR primers. Table S3. ChIP‐qPCR primers. Table S4. Buffer compositions. Table S5. Antibody information. Table S6. Differentially expressed genes in MDA‐MB‐231 (YAP1‐TEAD4 target genes). Table S7. Differentially expressed genes in HEK293 (YAP1‐TEAD1 target genes). Table S8. Differentially expressed genes in HEK293 (YAP1‐TEAD4 target genes). [file FEB2-600-370-s001.pdf]

Supplementary Table S1. Primers used in the vector construction

Gene ID	Forward primers (5' to 3')	Reverse primers (5' to 3')
YAP1	CAGGGTACCCAGAAGCCATGGATCCCG	CAGTCTAGATAACCATGTAAGAAAGCTTTCTT
TEAD1	CTAGGTACCACCGCCAAAATTGAGCCCA	CTAGAATTCTGTTCAGTCCTTTACAAGCCTG

Restriction enzyme sites that are used for cloning are underlined

Supplementary Table S2. RT-qPCR primers

Gene ID	Forward primers (5' to 3')	Reverse primers (5' to 3')	size (bp)
CTGF	GCGTGTGCACCGCCAAAGAT	CAGGGCTGGGCAGACGAACG	162
CYR61	AGCCTCGCATCCTATACAACC	TTCTTTCACAAGGCGGCACTC	143
SETDB1	GCCTACAGCAAGGAACGTATCC	GTTGATGGCAGGCACACTTGGA	121
GAPDH	GAAGGTGAAGGTCGGAGTCA	GTTGAGGTCAATGAAGGGGTC	118
AXL	CGTAACCTCCACCTGGTCTC	TCCCATCGTCTGACAGCA	121
ANKRD1	AGTAGAGGAACTGGTCACTGG	TGGGCTAGAAGTGTCTTCAGAT	138
ATF7IP	CTCTTCAACCATCTGGGGTG	TGGACGATGTGTTACATTCACG	98

Supplementary Table S3. ChIP-qPCR primers

Target	Forward primers (5' to 3')	Reverse primers (5' to 3')	size (bp)
CTGF _promoter	GGAGTGGTGCGAAGAGGATA	GCCAATGAGCTGAATGGAGT	235
CYR61 _promoter	TTGAGAATTCTGGAACGCGC	GTCTTTCGCTCGAGGTCCC	172
ANKRD1 _promoter	GAGGGGAGGACAAGCTAACC	CGATGTGATCACCACCAAAG	83

Supplementary Table S4. Buffer compositions

Buffer Name	Buffer composition	application
PBST	PBS with 0.05% Tween-20	IF
Permeabilization buffer	0.2% Triton X-100, PBST	IF
Blocking buffer (IF)	1% BSA, 22.52 mg/mL glycine, PBST	IF
Lysis buffer	1% NP-40, 50 mM Tris-HCl (pH 8.0), 150 mM NaCl, 2 mM EDTA (pH 8.0), protease inhibitors	WB, co-IP
TBST	TBS with 0.05% Tween-20	WB, co-IP
Blocking buffer (WB)	5% skim milk in TBST	WB, co-IP
ChIP lysis buffer	50 mM HEPES-KOH (pH 7.5), 140 mM NaCl, 1 mM EDTA (pH 8.0), 1% Triton X-100, 0.1% sodium deoxycholate, 0.1% SDS, protease inhibitors	ChIP
RIPA buffer	50 mM Tris-HCl (pH 8.0), 150 mM NaCl, 2 mM EDTA (pH8.0), 1% NP-40, 0.05% sodium deoxycholate, 0.1% SDS, protease inhibitors	ChIP
Blocking buffer (ChIP)	75 ng/uL single-stranded herring sperm DNA, 0.1 ug/uL BSA	ChIP
Low-salt wash buffer	20 mM Tris-HCl (pH 8.0), 150 mM NaCl, 2 mM EDTA, 1% Triton X-100, 0.1% SDS	ChIP
High-salt wash buffer	20 mM Tris-HCl (pH 8.0), 500 mM NaCl, 2 mM EDTA, 1% Triton X-100, 0.1% SDS	ChIP
LiCl wash buffer	10 mM Tris-HCl (pH 8.0), 250 mM LiCl, 1 mM EDTA, 1% NP-40, 1% sodium deoxycholate	ChIP
Elution buffer	100 mM $NaHCO_3$,1% SDS	ChIP

Supplementary Table S5. Antibody information

Antibody	Company	Catalog #	Host		Dilution
Anti-SETDB1	Proteintech	11231-1-AP	Rabbit	Polyclonal	1:2000 (WB), 1:300 (IF)
Anti-a-Tubulin	Santacruz	sc-23948	Mouse	Monoclonal	1:3000 (WB), 1:300 (IF)
Anti-DNMT1	Abcam	ab13537	Mouse	Monoclonal	1:2000 (WB)
Anti-ATF7IP	Bethyl	A300-169A	Rabbit	Polyclonal	1:2000 (WB), 1:200 (IF)
Anti-YAP1	CST	14074	Rabbit	Monoclonal	1:2000 (WB), 1:200 (IF)
Anti-p-YAP1(S127)	CST	4911	Rabbit	Polyclonal	1:2000 (WB)
Anti-GAPDH	CST	2118	Rabbit	Monoclonal	1:3000 (WB)
Anti-AKT	CST	9272	Rabbit	Polyclonal	1:2000 (WB)
Anti-p-AKT(T308)	CST	9275	Rabbit	Polyclonal	1:2000 (WB)
Anti-FLAG	Sigma	F3165	Mouse	Monoclonal	1:3000 (WB)
Anti-FLAG, HRP-linked	Abcam	ab49763	Mouse	Monoclonal	1:5000 (WB)
Anti-HA, HRP-linked	Roche	12013819001	Rat	Monoclonal	1:5000 (WB)
Anti-Rabbit IgG, HRP-linked	CST	7074	Goat	Polyclonal	1:5000 (WB)
Anti-Mouse IgG, HRP-linked	CST	7076	Horse	Polyclonal	1:5000 (WB)
Normal Rabbit IgG	CST	2729	Rabbit	Polyclonal	
Normal Mouse IgG	CST	68860	Mouse	Polyclonal	

Supplementary Table S6. Differentially expressed genes in MDA-MB-231 (YAP1-TEAD4 target genes)

Gene	WT vs KO1	WT vs KO2	WT vs KO3	Average	KO1 p-value	KO2 p-value	KO3 p-value
MISP	9.849200812	10.739048	10.43960517	10.34261799	8.71E-17	1.10E-19	1.11E-18
ITGBL1	5.075575323	7.524985967	8.449577264	7.016712851	0.000100903	1.94E-09	1.37E-11
NXPH2	5.634404638	6.930543544	5.964260132	6.176402771	2.51E-05	1.23E-07	6.83E-06
SLC7A10	3.977761813	5.734309434	4.943839924	4.885303724	0.010138618	8.86E-05	0.000888806
WWC3	3.052088419	5.337510687	5.9643416	4.784646902	6.80E-06	8.68E-17	1.05E-20
CLDN6	2.546203472	6.035270589	5.274983239	4.6188191	0.017150246	1.20E-09	1.26E-07
ANKRD1	1.696829788	4.02282509	5.611996403	3.777217094	0.015021898	1.36E-10	9.72E-20
SLCO1A2	3.050523149	3.95888846	3.739787656	3.583066422	0.007773133	0.000327924	0.000754771
THBS1	3.539683221	3.292201571	3.446210099	3.42603163	0.000128308	0.000362487	0.000187934
CTGF	2.017629974	3.274815457	4.583582463	3.292009298	5.02E-06	1.25E-14	9.02E-28
AXL	2.704205817	3.354541086	3.287957894	3.115568266	0.036278151	0.007260775	0.008847281
DUSP23	2.829132961	3.198387159	2.514160065	2.847226728	0.000308257	2.88E-05	0.001427008
S100A2	2.839324903	1.510537468	3.048008196	2.465956856	0.021220389	0.251175606	0.01191811
RBM24	2.127453483	1.890232609	3.151717272	2.389801121	0.002508952	0.007101579	3.26E-06
HRH1	2.041038244	1.767939173	3.006574454	2.271850624	0.049752014	0.088791829	0.002057474
FAM46B	2.576879896	2.132558534	2.092561071	2.267333167	1.54E-05	0.000368581	0.000512528
TSKS	2.178041746	2.542005614	1.959021311	2.226356224	0.001174282	8.98E-05	0.003554342
MYEOV	2.234566529	2.414490159	1.993598996	2.214218562	0.047811038	0.028689404	0.078312549
LMCD1	1.902983242	1.919581819	2.607684191	2.143416417	4.28E-07	2.68E-07	1.57E-12
KRT8	0.628209743	2.368944791	2.803186898	1.933447144	1.88E-06	1.23E-85	2.10E-120
DKK1	0.764052853	1.961017826	2.376015381	1.70036202	0.018608486	5.10E-11	9.91E-16
KCNJ2	1.394142655	1.37326888	2.250839704	1.672750413	0.020690818	0.019567773	5.47E-05
SOWAHD	1.201115658	1.456104375	1.765479626	1.47423322	0.048783289	0.012917449	0.00229156
DKK2	1.7510345	1.503778563	0.842837606	1.365883556	0.000985841	0.00456238	0.131274533
BNC1	1.259010063	1.505006756	1.188758971	1.31759193	0.001168004	7.97E-05	0.00211508
CCDC80	1.146330075	1.195607766	1.595294709	1.31241085	0.000184885	8.38E-05	1.33E-07
NUDT17	0.969904049	1.494655075	1.298424288	1.254327804	0.000169621	1.06E-09	1.89E-07
BEX1	-3.94132164	3.876606817	3.706918146	1.214067774	3.21E-10	1.21E-71	3.23E-65
TNXB	-1.48382115	2.34835984	2.770899831	1.211812841	0.036128695	5.54E-07	2.72E-09
CYR61	1.294373154	0.971396968	1.319883121	1.195217748	1.37E-07	7.75E-05	6.95E-08
HCP5	1.142354461	1.26763079	0.928439611	1.112808287	4.15E-06	2.01E-07	0.000190126
ENC1	0.867940175	0.905407939	1.533530095	1.102292736	1.43E-06	2.97E-07	6.93E-19
CCDC88B	0.973900063	1.114505244	0.995170692	1.027858666	0.0010506	0.000148867	0.000769291
LEPR	-0.81824647	-1.1651697	-1.05528097	-1.01289905	2.70E-06	2.58E-11	1.63E-09
PKP2	-0.63778092	-1.22320452	-1.22033212	-1.02710585	0.001753445	3.04E-09	4.17E-09
HIST1H3B	-1.2270241	-0.87884822	-1.05195838	-1.05261024	6.89E-08	7.78E-05	2.85E-06
ATP6V1C2	-1.23255133	-1.2341165	-0.90026032	-1.12230938	0.001204927	0.000981293	0.015223223
NOG	-1.8609678	-5.23865848	-4.31681916	-3.80548181	4.68E-06	5.00E-18	5.81E-17

Supplementary Table S7. Differentially expressed genes in HEK293 (YAP1-TEAD1 target genes)

Gene	WT vs KO1	WT vs KO2	WT vs KO3	Average	KO1 p-value	KO2 p-value	KO3 p-value
PEG10	8.03998108	10.2143302	9.08219717	9.11216949	1.2907E-53	9.3604E-86	4.2129E-68
FBXL7	5.13255961	8.69757055	10.6879554	8.17269519	5.637E-05	9.4979E-13	1.4543E-18
LY6K	5.28043824	5.65023713	6.10733416	5.67933651	6.5544E-07	8.5498E-08	6.581E-09
KCND2	4.30995161	5.90435871	5.06665028	5.09365353	0.00146758	7.4306E-06	0.00014093
CGREF1	4.28373814	4.87919403	5.00169163	4.72154127	1.4924E-14	7.5073E-19	1.013E-19
CLDN6	2.54620347	6.03527059	5.27498324	4.6188191	0.01715025	1.1988E-09	1.2563E-07
TEX19	5.25130631	4.27501693	4.06698725	4.5311035	1.1759E-41	7.4492E-28	3.7979E-25
ANKRD1	1.69682979	4.02282509	5.6119964	3.77721709	0.0150219	1.3598E-10	9.7217E-20
CTGF	2.01762997	3.27481546	4.58358246	3.2920093	5.021E-06	1.2522E-14	9.017E-28
C19orf38	2.89842472	2.98022583	3.0470418	2.97523078	5.7599E-07	2.1047E-07	1.1812E-07
GPSM3	2.40506297	2.21862824	3.87482681	2.83283934	0.00072419	0.00170135	6.123E-09
NKAIN2	2.22989325	2.53861785	2.77936727	2.51595946	0.00061917	5.7252E-05	9.3562E-06
GPRC5A	1.78127087	2.16555247	2.10860794	2.01847709	2.2623E-05	1.3193E-07	3.2855E-07
FAM83H	1.50945556	2.43591412	2.05188374	1.99908447	3.1817E-18	7.3687E-48	1.0709E-33
CLU	0.78745067	2.09269584	3.07851818	1.98622156	1.2942E-06	4.3407E-43	5.493E-94
ZNF234	1.61609661	1.88240008	2.05367819	1.85072496	8.0078E-22	4.6891E-30	1.7253E-35
DKK1	0.76405285	1.96101783	2.37601538	1.70036202	0.01860849	5.0955E-11	9.9139E-16
HSF2BP	1.70890762	1.94835841	1.23189597	1.62972066	0.00193505	0.00032251	0.0274676
F8	1.15887066	2.00053369	1.53453148	1.56464528	0.0090891	3.0587E-06	0.00042778
PLCL1	0.7444972	1.84609123	1.99083326	1.52714056	0.04982362	1.6539E-07	1.6486E-08
ACTRT3	0.73574855	2.09571102	1.71000271	1.51382076	0.00714414	4.6334E-18	5.6655E-12
SOWAHC	0.72280158	1.77995577	1.69124226	1.39799987	0.00025535	2.8385E-21	3.8491E-19
IRS1	1.09780921	1.45681168	1.27157266	1.27539785	3.2281E-05	1.766E-08	1.1185E-06
HEXIM1	1.50369552	0.97674729	1.28451885	1.25498722	1.093E-31	3.5658E-14	1.6811E-23
HOXA7	0.79821569	1.78459927	1.08798577	1.22360024	0.03175142	4.741E-07	0.00280032
CYR61	1.29437315	0.97139697	1.31988312	1.19521775	1.3685E-07	7.7539E-05	6.9546E-08
FAM26E	-2.1964962	2.33233103	3.39049921	1.17544469	0.01137681	2.9906E-06	4.7817E-12
HOXA6	0.44234998	2.06491653	1.01203052	1.17309901	0.00083026	5.826E-67	7.3312E-16
LRRC27	1.15945644	1.1420333	1.08909088	1.13019354	0.00181558	0.00197853	0.0033365
ENC1	0.86794018	0.90540794	1.5335301	1.10229274	1.4345E-06	2.974E-07	6.9334E-19
ID3	1.06113007	1.26289499	0.93815399	1.08739302	3.884E-23	7.9969E-33	1.9048E-18
RPH3AL	1.41088384	0.97093246	0.8184244	1.0667469	9.6684E-06	0.00244224	0.01151968
COL1A1	0.56503857	1.3563637	1.19467017	1.03869081	0.00126968	1.835E-15	3.3971E-12
POLR2A	1.34172635	0.75048303	0.98338033	1.02519657	4.572E-125	6.1379E-40	1.7148E-67
IGFBP5	0.39611929	-2.128386	-1.3846842	-1.0389836	0.00050493	4.0917E-47	2.2897E-25
HIST1H3B	-1.2270241	-0.8788482	-1.0519584	-1.0526102	6.894E-08	7.7781E-05	2.8521E-06
HIST2H2AC	-0.7038671	-0.8019816	-1.7745564	-1.0934684	0.00942117	0.00285594	5.3962E-10
TARBP1	-0.3056809	-1.458781	-1.7450156	-1.1698258	0.00021925	9.376E-65	2.5147E-87
ARMC4	-1.2810689	-1.2026533	-1.1186225	-1.2007816	1.7009E-08	3.6309E-08	3.2011E-07
DHRS3	-1.9412972	-1.2553277	-0.768766	-1.321797	1.7601E-22	2.8677E-12	1.125E-05
DOPEY2	-1.7307197	-1.3851221	-1.2947777	-1.4702065	3.3445E-20	2.0241E-15	1.2659E-13

NPTX1	0.35024747	-3.0413893	-2.2308322	-1.640658	0.00015114	8.256E-148	4.2689E-96
KCNQ4	-0.6469225	-1.9705448	-2.7379649	-1.7851441	0.04314359	1.0268E-08	5.4314E-13
PPP1R14C	-1.5640395	-2.5542349	-1.5318764	-1.8833836	7.9689E-24	7.4246E-53	1.2412E-23

Supplementary Table S8. Differentially expressed genes in HEK293 (YAP1-TEAD4 target genes)

Gene	WT vs KO1	WT vs KO2	WT vs KO3	Average	KO1 p-value	KO2 p-value	KO3 p-value
PEG10	8.03998108	10.2143302	9.08219717	9.11216949	1.2907E-53	9.3604E-86	4.2129E-68
FBXL7	5.13255961	8.69757055	10.6879554	8.17269519	5.637E-05	9.4979E-13	1.4543E-18
CGREF1	4.28373814	4.87919403	5.00169163	4.72154127	1.4924E-14	7.5073E-19	1.013E-19
CLDN6	2.54620347	6.03527059	5.27498324	4.6188191	0.01715025	1.1988E-09	1.2563E-07
TEX19	5.25130631	4.27501693	4.06698725	4.5311035	1.1759E-41	7.4492E-28	3.7979E-25
ANKRD1	1.69682979	4.02282509	5.6119964	3.77721709	0.0150219	1.3598E-10	9.7217E-20
XKR9	3.7157432	3.36088368	2.85492811	3.31051833	4.2732E-06	3.3152E-05	0.00055888
CTGF	2.01762997	3.27481546	4.58358246	3.2920093	5.021E-06	1.2522E-14	9.017E-28
SCN3B	2.21476542	3.44621832	3.4110064	3.02399671	1.9168E-07	7.6923E-18	2.2173E-17
GPSM3	2.40506297	2.21862824	3.87482681	2.83283934	0.00072419	0.00170135	6.123E-09
NKAIN2	2.22989325	2.53861785	2.77936727	2.51595946	0.00061917	5.7252E-05	9.3562E-06
MAFA	2.6575821	1.95990554	1.97871467	2.1987341	1.4994E-14	2.1573E-08	1.8195E-08
HIST1H3E	1.67437662	2.12852923	2.63095234	2.1446194	0.000461	3.2687E-06	5.0776E-09
LMCD1	1.90298324	1.91958182	2.60768419	2.14341642	4.2808E-07	2.6813E-07	1.5689E-12
ITGA9	1.45049896	2.06197083	2.55603098	2.02283359	0.0003232	8.0948E-08	1.6123E-11
FAM83H	1.50945556	2.43591412	2.05188374	1.99908447	3.1817E-18	7.3687E-48	1.0709E-33
CLU	0.78745067	2.09269584	3.07851818	1.98622156	1.2942E-06	4.3407E-43	5.493E-94
ZNF239	1.75296128	1.91399301	1.91771822	1.8615575	4.8688E-36	2.8433E-44	5.2499E-44
ZNF234	1.61609661	1.88240008	2.05367819	1.85072496	8.0078E-22	4.6891E-30	1.7253E-35
SYCE2	1.87861396	1.6677245	1.80811918	1.78481921	2.6277E-05	0.00018242	4.9788E-05
SCIN	1.18826124	2.50471749	1.51217378	1.73505084	0.03200513	7.7133E-07	0.00460971
DKK1	0.76405285	1.96101783	2.37601538	1.70036202	0.01860849	5.0955E-11	9.9139E-16
ZFP90	1.57472523	1.63632656	1.85834341	1.6897984	8.408E-75	2.8628E-82	6.584E-106
HSF2BP	1.70890762	1.94835841	1.23189597	1.62972066	0.00193505	0.00032251	0.0274676
F8	1.15887066	2.00053369	1.53453148	1.56464528	0.0090891	3.0587E-06	0.00042778
PLCL1	0.7444972	1.84609123	1.99083326	1.52714056	0.04982362	1.6539E-07	1.6486E-08
ACTRT3	0.73574855	2.09571102	1.71000271	1.51382076	0.00714414	4.6334E-18	5.6655E-12
RAD9B	1.3795954	1.14489333	1.77525736	1.43324869	0.00164961	0.00872862	2.6751E-05
SOWAHC	0.72280158	1.77995577	1.69124226	1.39799987	0.00025535	2.8385E-21	3.8491E-19
C10orf10	1.12961703	0.93359942	1.9731202	1.34544555	2.1386E-05	0.000424	2.434E-14
IRS1	1.09780921	1.45681168	1.27157266	1.27539785	3.2281E-05	1.766E-08	1.1185E-06
HEXIM1	1.50369552	0.97674729	1.28451885	1.25498722	1.093E-31	3.5658E-14	1.6811E-23
TFPI2	0.97624321	1.2757571	1.47482655	1.24227562	0.00255915	4.5166E-05	2.2328E-06
ZNF695	0.74574804	1.33053345	1.64048829	1.23892326	6.6475E-06	1.6483E-17	3.2525E-26
HOXA7	0.79821569	1.78459927	1.08798577	1.22360024	0.03175142	4.741E-07	0.00280032
CYR61	1.29437315	0.97139697	1.31988312	1.19521775	1.3685E-07	7.7539E-05	6.9546E-08
FAM26E	-2.1964962	2.33233103	3.39049921	1.17544469	0.01137681	2.9906E-06	4.7817E-12
HOXA6	0.44234998	2.06491653	1.01203052	1.17309901	0.00083026	5.826E-67	7.3312E-16
LRRC27	1.15945644	1.1420333	1.08909088	1.13019354	0.00181558	0.00197853	0.0033365
TDRP	1.53442264	1.08734814	0.76813648	1.12996909	2.7031E-12	8.3993E-07	0.00065439
CDKN2B	1.014336	0.86097748	1.51140966	1.12890772	4.145E-09	5.2425E-07	3.5121E-19

ZNF805	0.74735038	1.04356085	1.52682726	1.10591283	0.00234295	1.4039E-05	1.4288E-10
ID3	1.06113007	1.26289499	0.93815399	1.08739302	3.884E-23	7.9969E-33	1.9048E-18
ZNF44	0.944411	1.08861692	1.12637766	1.05313519	7.3975E-05	3.2416E-06	1.6109E-06
COL1A1	0.56503857	1.3563637	1.19467017	1.03869081	0.00126968	1.835E-15	3.3971E-12
POLR2A	1.34172635	0.75048303	0.98338033	1.02519657	4.572E-125	6.1379E-40	1.7148E-67
IGFBP5	0.39611929	-2.128386	-1.3846842	-1.0389836	0.00050493	4.0917E-47	2.2897E-25
STAMBPL1	-1.1832239	-1.0413812	-0.9173539	-1.0473197	1.088E-06	7.6601E-06	7.9876E-05
HIST1H3B	-1.2270241	-0.8788482	-1.0519584	-1.0526102	6.894E-08	7.7781E-05	2.8521E-06
SH2D5	-0.4404328	-0.7863241	-1.9384946	-1.0550838	0.00503895	6.2313E-07	2.6296E-26
CASZ1	-0.572774	-1.6191177	-1.0534114	-1.0817677	0.02033459	5.6807E-10	2.9159E-05
HIST2H2AC	-0.7038671	-0.8019816	-1.7745564	-1.0934684	0.00942117	0.00285594	5.3962E-10
TARBP1	-0.3056809	-1.458781	-1.7450156	-1.1698258	0.00021925	9.376E-65	2.5147E-87
ARMC4	-1.2810689	-1.2026533	-1.1186225	-1.2007816	1.7009E-08	3.6309E-08	3.2011E-07
ICAM5	-0.9424768	-1.4361474	-1.3279818	-1.2355353	3.6575E-09	7.3594E-19	2.5881E-16
CPNE1	-0.8413606	-1.5706051	-1.3123407	-1.2414355	4.4308E-19	9.1238E-60	8.5118E-43
GPM6A	-1.3396222	-1.0935493	-1.2972501	-1.2434739	7.4622E-08	6.8363E-06	1.4452E-07
HILPDA	-0.4389144	-1.4133635	-1.9503621	-1.2675466	0.02155927	2.2733E-13	2.1134E-23
DHRS3	-1.9412972	-1.2553277	-0.768766	-1.321797	1.7601E-22	2.8677E-12	1.125E-05
NHSL1	-1.8103602	-1.3286561	-0.8719418	-1.336986	9.0934E-13	4.3984E-08	0.00027587
NOS1AP	-1.0944288	-1.9187934	-1.1227125	-1.3786449	0.04413667	0.00057071	0.03811745
FGFBP3	-1.2390585	-2.1888967	-1.6734366	-1.7004639	2.5907E-05	2.7659E-11	3.9992E-08
LY6G6D	-4.6859946	1.8169557	-2.5248577	-1.7979655	0.00020432	0.00020014	0.00815448
FERMT1	-1.5644271	-1.5244737	-2.4208524	-1.8365844	2.054E-05	1.4908E-05	2.4841E-09
PPP1R14C	-1.5640395	-2.5542349	-1.5318764	-1.8833836	7.9689E-24	7.4246E-53	1.2412E-23
PODXL	-0.6240558	-2.3834374	-2.7583529	-1.9219487	0.00051763	8.9946E-35	1.9982E-42
SNTB1	-1.7821811	-2.1486442	-2.076898	-2.0025744	4.7842E-10	9.5773E-14	7.3717E-13
LRRC26	-3.3606799	-3.652791	-3.551256	-3.5215756	0.0245349	0.01452184	0.0169157
LYPD1	-4.0183758	-6.5098611	-5.0413997	-5.1898789	1.3448E-07	1.7027E-07	7.8994E-08
